# Interleukin-17 Could Promote Breast Cancer Progression at Several Stages of the Disease

**DOI:** 10.1155/2015/804347

**Published:** 2015-12-13

**Authors:** Thomas Welte, Xiang H.-F. Zhang

**Affiliations:** ^1^Diana Helis Henry Medical Research Foundation, New Orleans, LA 70130, USA; ^2^Lester and Sue Smith Breast Center, Baylor College of Medicine, One Baylor Plaza, Houston, TX 77030, USA; ^3^Dan L. Duncan Cancer Center, Baylor College of Medicine, One Baylor Plaza, Houston, TX 77030, USA; ^4^Department of Molecular and Cellular Biology, Baylor College of Medicine, One Baylor Plaza, Houston, TX 77030, USA; ^5^McNair Medical Institute, Baylor College of Medicine, One Baylor Plaza, Houston, TX 77030, USA

## Abstract

Metastatic disease accounts for more than 90% of deaths from breast cancer. Yet the factors that trigger metastasis, often years after primary tumor removal, are not understood well. Recently the proinflammatory cytokine interleukin- (IL-) 17 family has been associated with poor prognosis in breast cancer. Here we review current literature on the pathogenic mechanisms driven by IL-17 during breast cancer progression and connect these findings to metastasis. These include (1) direct effects of IL-17 on tumor cells promoting tumor cell survival and invasiveness, (2) regulation of tumor angiogenesis, and (3) interaction with myeloid derived suppressor cells (MDSCs) to inhibit antitumor immune response and collaborate at the distant metastatic site. Furthermore, IL-17 might also be a culprit in bone destruction caused by late stage bone metastasis. Interestingly, in addition to these potential prometastasis functions, there is also evidence for an opposite, antitumor role of IL-17 during cancer therapies. We hypothesize that these contradictory roles may be due to chronic, imbalanced versus acute transient nature of the immune reactions, as well as differences in the cells that interact with IL-17^+^ cells under different circumstances.

## 1. Introduction

Although great progress has been made in breast cancer therapy, the treatments are often unsuccessful once metastases to vital organs occur. After removal of the primary breast tumor, the disease can be dormant over several years. As of today we are unable to predict reliably if or when the cancer will progress. Therefore it is important to elucidate pathogenic factors involved. IL-17 is a proinflammatory cytokine family with a documented association with poor prognosis in breast cancer. In our review, we discuss recent findings on IL-17-driven mechanisms that promote breast cancer progression. We also attempt to discern the context in which IL-17 has the opposite role and mediates antitumor reactions during cancer therapies.

## 2. IL-17 Family

The IL-17 family is comprised of 6 cytokines, including IL-17A to IL-17F. Among them, IL-17A and IL-17F share the highest sequence homology and have similar biological functions. Both bind to IL-17RA and IL-17RC chains [1]. IL-17 is primarily secreted by T helper (Th) 17 cells and innate lymphocytes (*γδ* T cells, natural killer (NK) cells, and innate lymphoid cells) [2]. The prototypic IL-17A induces a signaling cascade in its target cells by binding to its receptor IL-17RA/RC. These receptor chains are broadly expressed in many cell types accounting for the pleiotropic effects of IL-17A on epithelial cells (including transformed tumor cells), endothelial cells, osteoblasts, fibroblasts, and various myeloid cells [3]. Downstream of the IL-17 receptor, NF*κ*B activator 1 (Act1) and TNF receptor associated factor (TRAF) 4 are important adaptor proteins that transmit the intracellular signal cascade to activate transcription factors such as nuclear factor kappa B (NF*κ*B) and activator protein (AP) 1 in cell and tissue-specific manner. In turn these transcription factors regulate a wide array of target genes.

IL-17 induces expression of certain chemokines and vascular endothelial growth factor (VEGF) that lead to the recruitment of specific subsets of immune cells to the site of inflammation and the induction of angiogenesis, respectively. Loss of control in IL-17 signaling is a common pathogenic mechanism in chronic inflammatory diseases/autoimmune diseases such as rheumatoid arthritis, psoriasis, and inflammatory bowel disease [4]. In rheumatoid arthritis, IL-17 induces the expression of receptor activator of nuclear factor kappa B ligand (RANKL) in osteoblasts which leads to the activation of osteoclasts. In addition among other IL-17 target genes, matrix metalloproteinases (MMPs) and IL-6 play important roles in bone resorption and pathogenesis of the disease [4]. Although IL-17A/F is typically associated with destructive tissue damage in autoimmune disease and pathogen infections [5–7], it is also involved in protective immunity. For example, IL-17A acts as a potent inducer of T cell mediated immune responses by activating and recruiting dendritic cells (DCs), monocytes, and neutrophils in various tissues [2]. The recruitment and modulation of neutrophils are critical in host defense [8, 9]. However, beyond certain pathogen infection, IL-17 has an almost ubiquitous role as coordinator of neutrophil-dominated inflammation. Furthermore, IL-17 stimulates dendritic cells (DCs) to produce IL-12, while inhibiting IL-10 to augment Th1 differentiation [10, 11].

## 3. Role of IL-17 in Breast Cancer

### 3.1. Presence of IL-17 in Breast Cancer Patients

Studies on tumor infiltrating lymphocytes (TILs) of breast cancer patients revealed the presence of Th17 cells and pointed out the role of tumor microenvironmental factors including chemokine (C-C motif) ligand 5 and monocyte chemoattractant protein 1 in generating and attracting these cells into the tumors [12]. IL-17^+^ immune cells (either lymphocytes or macrophages) were also detected by Cochaud et al. in TILs of 8 of 40 breast cancer patients [13]. Furthermore, while analyzing 207 breast cancer specimens by IL-17 immunohistochemistry, Chen et al. found a correlation between large numbers of IL-17^+^ cells and high histological grade of the tumors, triple negative molecular subtype, and shorter disease-free survival [14]. Another study showed that three single nucleotide polymorphisms (SNPs) in the IL-17A gene are associated with increased breast cancer risk in a Chinese patient population [15]. Studying invasive ductal carcinoma (IDC) of the breast, Benevides et al. determined an association between coordinately upregulated T_reg_ and Th17 cells and aggressiveness of the disease [16, 17]. These and other findings discussed below in Sections 3.2 to 3.5 are in support of IL-17 as a marker of poor prognosis and risk in breast cancer. However, it was also recognized that the blood of metastatic breast cancer patients contains a higher frequency of T_reg_ cells associated with risk. And in the HER2^+^ subtype of breast cancer very low levels of Th17 cells were observed with an inverse relationship between T_reg_ cells and Th17 cells. Anti-HER2 (trastuzumab) treatment increased Th17 cell numbers to restore a balance between T_reg_ and Th17 cells [18]. Similarly, other IL-17 family members have been correlated to pro- and antibreast cancer activities [19–25]. Therefore it is necessary to investigate the detailed mechanisms to decipher IL-17's multiple roles in breast cancer.

### 3.2. Direct Effects of IL-17 on Breast Cancer Cells

Due to the high frequency of expression of IL-17 receptor chains on tumor cells, IL-17 family members can have direct effects on tumor cells. In a mouse breast cancer model, tumor-induced transforming growth factor beta (TGF-*β*) induced CD8 T cells to produce IL-17.* In vitro*, IL-17 in turn suppressed apoptosis of 4T1 mouse mammary carcinoma, CT26 mouse colon carcinoma, and MDA-MB231 human breast carcinoma cell lines. Furthermore, IL-17R knockdown in 4T1 breast cancer cells enhanced apoptosis and decreased tumor growth* in vivo* [26]. Treatment with endothelin-1 receptor dual antagonist decreased IL-17A levels and caused slower 4T1 tumor growth [27]. In another study, IL-17A via activation of tumor progression locus 2 (TPL2) induced mitogen activated protein kinase (MAPK), c-jun N-terminal kinase (JNK), and signal transducer and activator of transcription (STAT) 3 signaling and cellular transformation in JB6 CI41 mouse epidermal cells and promoted MCF7 (human breast cancer cell line) tumorigenicity [28]. MDA-MB231 cells express CD40 and interact with CD40L on activated T cells. This interaction increased TGF-*β* production and consequently induced IL-17 expression, which enhanced MDA-MB231 proliferation through STAT3 activation [29]. Furthermore, IL-17A and IL-17E (IL-25) were involved in proliferation and survival of human breast cancer cell lines T47D and MCF7 as well as primary breast cancer biopsy cells IJG1731 and thereby promoted their resistance to the antimitotic chemotherapy agent docetaxel [19]. Interestingly, under some circumstances, IL-17E (IL-25) produced by nonmalignant mammary epithelial cells also displays antitumor function by targeting adjacent tumor cells, which express high levels of IL-25R and inducing apoptosis [20]. Similar to TNF-*α* receptor, a death domain (DD) portion in the IL-25R may be linked to apoptosis inducing adaptor molecules FAS-associated protein with death domain (FADD) and TNF-R1-associated death domain protein (TRADD) under certain conditions. These conclusions are also supported by [21]. IL-17 also plays a role in tumor invasion. For example, cells in the peritumoral area expressed IL-17 in 8 of 19 breast cancer patients studied.* In vitro*, the human breast cancer cell lines MDA-MB231 and MDA-MB435 were examined in a matrigel invasion assay by plating them on matrigel invasion chambers. Added IL-17 greatly induced invasion of these cells into matrigel [30].

### 3.3. IL-17 in Angiogenesis

It has been found that the tumor-promoting IL-17: tumor angiogenesis axis causes resistance to anti-VEGF therapy [31]. Tumor infiltrating Th17 cells and IL-17 induced the expression of granulocyte-colony stimulating factor (G-CSF) leading to immature myeloid cell recruitment into the tumor microenvironment and anti-VEGF therapy resistance through the production of proangiogenic Bv8 by these myeloid cells [31]. In patients with invasive ductal carcinoma (IDC), tumor aggressiveness was reported to be enhanced by IL-17 via induction of angiogenic factors, such as chemokine CXCL8, MMP-2, MMP-9, and VEGF [16, 32]. Similarly, injection of recombinant IL-17 in the murine breast cancer model 4T1 was shown to increase microvascular density, a parameter for tumor angiogenesis [33].

### 3.4. IL-17 and Tumor Escape from Host Immunosurveillance

**Figure 1:**
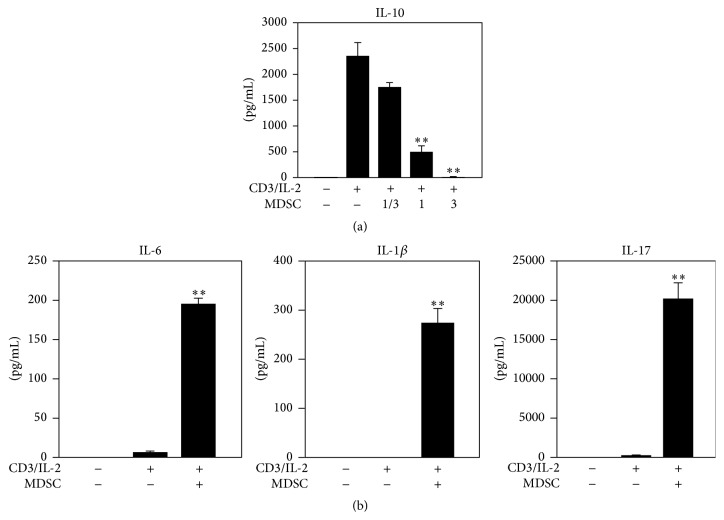
The effects of MDSCs on cytokine expression of T cells in coculture. Bio-Plex assays of indicated cytokines are shown. MDSCs and T cells were admixed at three ratios: 0.33 : 1, 1 : 1, and 3 : 1 (a) or 1 : 1 ratio (b) for five days. (a) Inhibited by MDSC coculture. (b) Cytokines of Th17 formation axis. *n* = 4. ^*∗∗*^ *p* < 0.01.

In an attempt to produce a specific antihuman cancer vaccine by combining one of the most specific cancer-associated structures, the Tn antigen (alpha-GalNAC-O-Ser/Thr) with mucin (MUC) 6, it was noted that Tn strongly diminished immunogenicity compared to MUC6 alone via a partial abrogation of Th1 response and promotion of IL-17 responses [34]. Other studies further revealed the underlying mechanism of IL-17's immunosuppressive function. In polyoma middle T- (PyMT-) induced breast cancer models, IL-17 induced the secretion of CXCL1 and CXCL5 from mammary carcinoma cells and increased the suppressive function of myeloid derived suppressor cells (MDSCs) on T cells by upregulating arginase (Arg), indoleamine 2,3-dioxygenase (IDO), and prostaglandin- endoperoxide synthase- (COX-) 2 expression [35]. The connection between breast cancer cells, IL-17, and MDSCs was further elucidated by Coffelt et al. [36] while studying a different model of breast cancer (tissue-specific cadherin (Cdh) 1/p53 double knockout). The tumor-derived IL-1*β* induced IL-17 expression by *γδ* T cells, which resulted in G-CSF-dependent expansion and polarization of neutrophils into cells with MDSC characteristics. These polarized neutrophils further inhibited cytotoxic CD8 T cells, which otherwise limited metastasis [36]. Thus, the collaboration of IL-17^+^ *γδ* T cells with MDSCs is important for tumor escape from host immune reactions and metastasis formation in distant organs. In an attempt to understand the interactions of T cells and MDSCs in breast cancer models (Welte et al., submitted), we confirmed the immunosuppressive activities of breast tumor-induced MDSCs (Welte et al., submitted) in the* in vitro* coculture assay. MDSCs isolated from blood of breast tumor bearing mice were admixed with naïve splenic T cells. Cocultures were stimulated with combination of plate-bound anti-CD3 and IL-2 and T cell proliferation was assessed by the CFSE-labeling method. Furthermore, cytokine secretion was compared to that of T cells activated in the absence of MDSCs. It was noted that the regulatory cytokine IL-10 was diminished in the presence of MDSCs (Figure 1(a)), suggesting an IL-10- independent inhibition on T cell proliferation. Interestingly, T cells cocultured with large numbers of MDSCs produced higher levels of IL-6, IL-1*β*, and IL-17, which indicates MDSCs promote Th17 cell differentiation (Figure 1(b)). In addition to Th17 cells, *γδ* T cells serve as one main resource for IL-17 [36, 37]. In breast cancer MDSCs promoted the tumor-infiltration of V*γ*4^+^CCR6^+^ T cells (Welte et al., unpublished), subsets phenotypically related to IL-17 producers in previous studies [38]. Overall, these findings suggest a complex reciprocal interaction between MDSCs and IL-17 producing T cells.

### 3.5. IL-17 and Breast Cancer Metastasis

In addition to the enhancement of metastasis via suppression of antitumor immunity, IL-17 may also promote metastasis through its inflammatory activities. Eiró et al. found a link between intratumoral MMP-11^+^ mononuclear inflammatory cells and metastasis [39, 40]. IL-17 was among the proinflammatory factors associated with MMP-11^+^ cells, although its function in these cells still needs to be demonstrated. A very recent report analyzed the pathogenesis mechanism of metastasis in invasive ductal carcinoma (IDA) and found that IL-17A affected different steps of metastasis such as migration of tumorigenic neutrophils and tumor cells to distant metastatic sites and production of IL-6 and CCL20 in metastatic tumor cells [17]. A previous study also found that the metastasis promoting interaction of human bone marrow-derived stem cells with breast cancer tumor cells could be mediated by IL-17B/IL-17BR signaling [41]. The role of IL-17 in breast cancer metastasis to bone is also demonstrated by studies in Mukherjee's lab. High levels of IL-17A are associated with autoimmune arthritis. Mukherjee's group generated arthritic mouse models and found enhanced bone and lung metastasis of breast cancer line 4T1 and PyMT mouse breast cancer model upon arthritis induction. Combination therapy of anti-IL-17 and anti-inflammatory celecoxib abrogated metastasis [42–44]. A severe side effect of cancer treatment with aromatase inhibitor is arthralgia mediated by IL-17 [45–47]. Based on these findings, it is an attractive hypothesis that bone-destructive events in autoimmune arthritis and at late stage bone metastasis could both be caused by an IL-17-dependent mechanism.

## 4. Summary and Future Perspective

Since the discoveries that identified IL-17 as a central proinflammatory mediator, it has garnered a lot of attention and our knowledge on IL-17 is expanding quickly. In the biology of many tumors, IL-17 cytokines have a protumor role either directly on tumor cells or indirectly by having detrimental effects on the patient's antitumor response and by causing microenvironmental changes that worsen the disease towards more invasive and metastatic phenotype. Furthermore, IL-17-induced changes in rheumatoid arthritis resemble the bone destruction phenotypes observed at late stage bone metastasis. Therefore, treatment with anti-IL-17 holds a promise as anticancer therapy and in ameliorating the effects of bone metastasis. One concern in this treatment is that it could make patients more vulnerable to infections normally held in check by IL-17. Antitumor activity of interferon (IFN) *γ* is well established. In comparison, it is less clear whether IL-17 could have beneficial effects in the immunotherapy of cancer. Recent studies by Zitvogel and colleagues described how certain chemotherapy drugs such as doxorubicin induce “immunogenic” tumor cell death [48, 49]. The immune response mounted to the dying tumor cells is critical for the efficacy of these chemotherapeutic drugs in preventing tumor progression. IL-17 expressed by *γδ* T17 cells is important for initiating this immune reaction and eventually leads to activation of protective tumor-specific IFN*γ* expressing CD8 T cells. The challenge in future studies will be to understand how to restrict IL-17's role to the promotion of CD8/IFN*γ* antitumor activity without unleashing its other pathogenic protumor functions described above.
